# Observation of Three-Photon Cascaded Emission from Triexcitons in Giant CsPbBr_3_ Quantum Dots at Room Temperature

**DOI:** 10.1021/acs.nanolett.4c03096

**Published:** 2024-10-14

**Authors:** Miri Kazes, Dekel Nakar, Ihor Cherniukh, Maryna I. Bodnarchuk, Leon G. Feld, Chenglian Zhu, Daniel Amgar, Gabriele Rainò, Maksym V. Kovalenko, Dan Oron

**Affiliations:** † Department of Molecular Chemistry and Materials Science, Weizmann Institute of Science, Rehovot 7610001, Israel; ‡ Laboratory for Thin Films and Photovoltaics, Empa, Swiss Federal Laboratories for Materials Science and Technology, 8600 Dübendorf, Switzerland; § Institute of Inorganic Chemistry, Department of Chemistry and Applied Biosciences, ETH Zürich, 8093 Zürich, Switzerland; ∥ National Centre of Competence in Research (NCCR) Catalysis, ETH Zürich, CH-8093 Zürich, Switzerland

**Keywords:** Perovskite nanocrystals, photon correlation, multiexcitons, photon cascades, single-particle spectroscopy, quantum dots

## Abstract

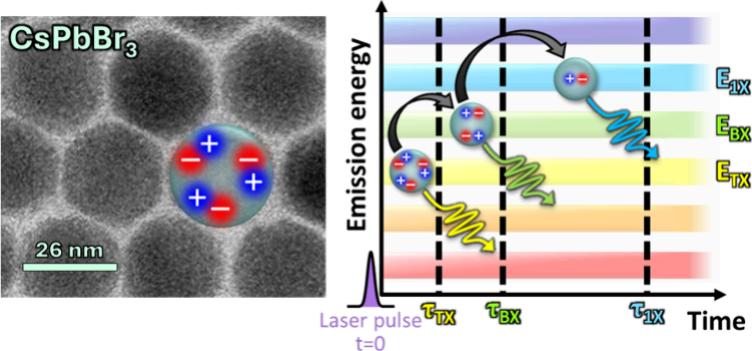

Colloidal semiconductor nanocrystals have long been considered a promising source of time-correlated and entangled photons via the cascaded emission of multiexcitonic states. The spectroscopy of such cascaded emission, however, is hindered by efficient nonradiative Auger-Meitner decay, rendering multiexcitonic states nonemissive. Here we present room-temperature heralded spectroscopy of three-photon cascades from triexcitons in giant CsPbBr_3_ nanocrystals. We show that this system exhibits second- and third-order correlation function values, g^(2)^(0) and g^(3)^(0,0), close to unity, identifying very weak binding of both biexcitons and triexcitons. Combining fluorescence lifetime analysis, photon statistics, and spectroscopy, we can readily identify emission from higher multiexcitonic states. We use this to verify emission from a single emitter despite high emission quantum yields of multiply excited states and comparable emission lifetimes of singly and multiply excited states. Finally, we present potential pathways toward control of the photon number statistics of multiexcitonic emission cascades.

Size confinement in quantum dots (QDs) gives rise to discrete atomic-like electronic states with sharp optical transitions.^1^ Absorption of a photon by a QD leads to the formation of an exciton, a bound electron–hole pair, which can then recombine through the emission of a photon. Under stronger optical pumping, multiexcitons can be generated—typically with lower probabilities as the number of absorbed photons follows a Poisson distribution function. Multiexcitons can decay by emitting photons in a cascaded manner, but this process is hindered by efficient nonradiative Auger decay, limiting the multiexciton-multiphoton emission quantum yield (QY). To date, multiexciton spectroscopy has mostly focused on the doubly excited (biexciton, BX) state. This is a result of both its importance in the contexts of optical gain and quantum-light emission and the relative simplicity of accessing it. Much less attention has been directed toward spectroscopy of higher excited states such as the triply excited (triexciton, TX) state. This is unsurprising, since the emission QY from higher excited states was typically considered to be very small, especially for QDs in the strong-confinement regime. Advances in the synthesis of large QDs, corresponding to the intermediate- or weak-confinement regime, where Auger recombination is significantly slowed down, opened a niche where cascaded emission from higher excited states becomes accessible, as demonstrated by early quasi-CW measurements of weakly confined CuBr QDs.^2^ One, however, faces related spectroscopic challenges. For example, identifying emission from higher excited states, often based on a large difference in the relaxation kinetics (or energy levels), has become more difficult. In fact, even the identification of single emitters, typically based on observing a second-order photon correlation function (g^(2)^) well below unity, has become difficult since g^(2)^, determined by the ratio of the QY of the BX relative to that of the single exciton state (1X),^3^ can approach unity in these systems.^4^

TX emission was measured early on in weakly confined CuCl QDs embedded in a NaCl matrix and later in molecular-beam epitaxy (MBE) grown InAs/GaAs QDs.^5,6^ In single colloidal nanocrystals, TX emission with a high QY was demonstrated before in core–shell CdSe/CdS NPLs and QDs where the large lateral dimension could accommodate three excitons with lower Auger-recombination rates.^7−9^

Perhaps the most natural manner in which higher excited states, specifically TXs, can be studied is by detection of cascaded emission. A cascaded radiative electron–hole recombination from the TX to the BX to the 1X and further to the ground state results in the emission of three photons in rapid succession. This was realized, for example, by Gershoni et al.,^10^ using a layer of self-assembled InGaAs QDs grown by MBE on top of an oriented GaAs substrate in a planar microcavity designed for enhancing emission light harvesting. There, a three-pulse sequence at the resonance was used to generate the TX state. This spectroscopic study of multiexcitonic states required cryogenic temperatures due to the small energy differences between states.^11^

To date, TX emission was investigated by a three-channel Hanbury Brown and Twiss setup, measuring the third-order photon correlation function (g^(3)^).^7,8^ However, this is an indirect measurement. Furthermore, in order to obtain spectral information, color filtering was used, but it cannot resolve weak interaction energies in multiexcitons.^9^ Notably, the limited number of detectors used in these experiments places significant limits on the detection efficiency, as multiple photons often end up in the same channel, and the triple-photon event is thus not efficiently measured.

The heralded spectroscopy technique, used here, utilizes a *SpectroSPAD* system, which includes a single-photon avalanche diode (SPAD) array at the output of a grating spectrometer.^12^ This system enabled the detection of cascaded BX–1X events in a temporally and spectrally resolved manner at room temperature, adding a spectral dimension to previous studies that utilized only temporal photon correlations. *SpectroSPAD* was used before to measure the room-temperature BX binding energy of single CdSe/CdS/ZnS,^13^ CsPbBr_3_,^14^ and CsPbI_3_ QDs.^14^

Lead halide perovskite QDs are promising candidates for the development of quantum-light sources operating even at room temperature, showing enhanced absorption cross-section, blinking-free photoluminescence (PL) with QYs close to unity, and narrow emission lines, superseding their II–VI and III–V QDs counterparts.^15−19^ Their high QY arises from the short radiative lifetime and the apparent absence of surface trap states.^20,21^ Recently, efficient emission from biexcitons was observed in perovskite QDs, motivating their exploration for multiphoton emission.^22,23^ Additionally, superbunching was observed in single CsPbBr_3_ QDs, caused by a cascaded BX–1X emission. This stems from a dark long-lived band-edge exciton state, which promotes the creation of BXs at low temperatures, enabling the emission of correlated photon pairs.^24^ To promote triple-cascaded emission from the TX-BX-1X excitonic states, we study giant CsPbBr_3_ QDs, several times the exciton Bohr diameter, benefiting from the typical brightness of this family of materials and a reduced Auger interaction due to weak confinement, increasing the emission QY of multiexcitonic states.

Giant CsPbBr_3_ QDs of about 26 nm in diameter were synthesized according to the procedure described in Supporting Information section S1. The ensemble emission measured in solution is centered at around 519 nm (2.39 eV) with an emission QY of 27.5%. Ensemble absorption and emission spectra, a transmission electron microscope (TEM) image, and size distribution can be found in Figure S1. Multiexciton emission is measured using heralded spectroscopy, a single-particle and single-photon sensitive correlation method that enables simultaneous detection of single photons with both time and energy resolution.^13^ Briefly, an inverted microscope with a high numerical aperture objective is used to focus a pulsed diode-laser illumination (PicoQuant, LDH–P-C-470B, 70 ps, 470 nm) on a single QD and collect emitted PL in the backward direction. To achieve sufficient count rates, the excitation power used was 40 nW, which is at the saturation power for these particles (Figure S2). PL is passed through a home-built Czerny–Turner spectrometer with a single-photon avalanche diode (SPAD) array detector, so that each detected photon is time-stamped according to its arrival time and energy-stamped according to the array pixel it was detected in (Figure 1). Full details on the SPAD array setup are given in Supporting Information section S2. Postselecting only trains of photons emitted following the same excitation pulse, and time-gating the cascade of multiexciton emission, robustly differentiates between the emission decay of the TX, BX, and other overlapping long-lived emitting states, such as 1X or trions. Since triple-photon events are still sparse in time, and to ensure sufficient statistics, we show below only measurements that yielded at least 2000 triple-photon events over the course of a 5 min measurement (24 out of ∼60 particles measured). In parallel, to validate our findings, we separately analyzed all pair-photon events.

**Figure 1:**
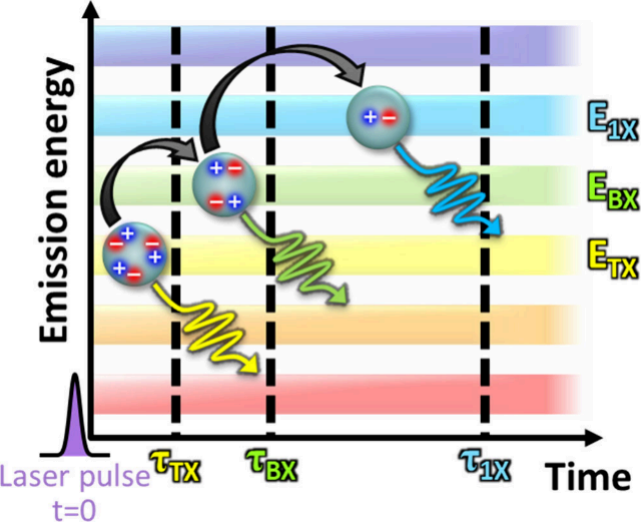
Scheme of cascaded TX to BX to 1X to ground-state emission in time and emission energy, as measured here by the heralded spectroscopy method. We note that while the color ordering (TX red-shifted from BX, which in turn is red-shifted from 1X) is characteristic of the system under study here, it is not representative of every QD system.

Figure 2a shows the normalized histogram of the photon counts according to their arrival time for triple-photon cascades. Details on data processing appear in Supporting Information section S3. To reduce artifacts due to interpixel crosstalk and dark counts in the SPAD array, we consider only detections within the following time gates: from −0.5 to 2 ns from the laser pulse for the first detected photon (TX), from 0.2 to 4 ns from the first detection for the second photon (BX), and from 0.5 to 20 ns from the second photon for the third photon (1X). These are plotted in blue, green, and yellow, respectively.

**Figure 2:**
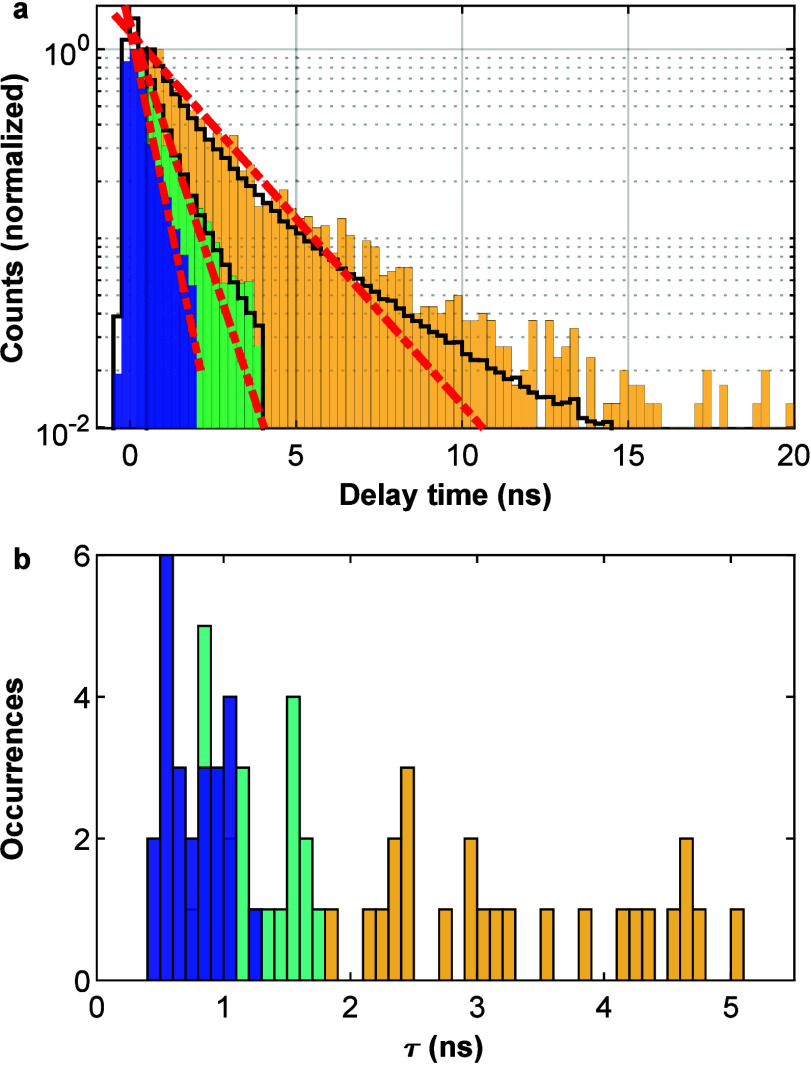
(a) Histogram of triple-cascaded photons from a single measurement, time-gated, and plotted according to their arrival time. TX, BX, and 1X photons marked in blue, green, and yellow bars, respectively. TX arrival times are relative to the excitation pulse, and BX and 1X arrival times are relative to the preceding photon detection. Red lines correspond to monoexponential decay fits. Black lines correspond to histograms taken from pair-photon analysis and show BX and 1X photons. (b) Ensemble histogram of emission decay coefficients calculated using a monoexponential fit over each of the emission decay curves in (a), from all analyzed QDs.

We first want to verify that the measured signals are free of artifacts caused by dark counts or interpixel crosstalk. To this end, we compare the histograms of photon-arrival times from triple-photon cascades with those obtained from pair cascades, as plotted in the black curves. The decays of the second photon in the triple cascade and the first photon in the double cascade are practically identical. The same is observed for the third photon in the triple cascade and the second photon in the double one. This agreement shows that the triple-cascade signal is indeed representative of the emission from the probed nanocrystal. We can therefore assign the decay components plotted in Figure 2a to a cascaded TX-BX-1X emission, assuming that the emission originates from a single particle, as we will show below. For this particular QD, we obtain lifetimes of τ_TX_ = 0.51 ± 0.08 ns (R^2^ = 0.99), τ_BX_ = 0.82 ± 0.11 ns (R^2^ = 0.98), and τ_1X_ = 2.21 ± 0.17 ns (R^2^ = 0.96) by fitting single exponential curves (dashed red lines). A similar analysis of such measurements from another particle is presented in the Supporting Information (Figure S3). A summary of extracted lifetimes of TX, BX, and 1X from triple-photon measurements of all measured particles is presented in Figure 2b. An increase in decay lifetime going from TX (blue) through BX (green) to 1X emission (yellow) is apparent with mean lifetimes of 0.78, 1.19, and 3.33 ns for TX, BX, and 1X emission, respectively. As discussed below, these lifetimes are dominated by the decay dynamics of the single exciton, radiative and nonradiative (since Auger recombination is rather weak for such large particles).^7^ We note that the ratio of mean TX to mean BX lifetimes is close to the 2:3 ratio of the number of excitons in the particle. The mean BX lifetime is about 2.8 times shorter than that of the 1X.

Resolving the QD emission not only in time but also in energy, spectra of the TX, BX, and 1X can be constructed and fitted to find their respective emission peak centers, as shown in Figure 3a, in blue, green, and yellow, respectively. The shift between the 1X peak and the higher-order excitonic peaks is a measure of the BX and TX binding energies (BEs). Here, BE is defined to be positive for attractive interactions (BE_BX_ ≡ E_1X_ – E_BX_ and BE_TX_ ≡ E_1X_ – E_TX_). For this QD, BE_TX_ = 0.99 ± 1.42 meV and BE_BX_ = 0.24 ± 1.42 meV were extracted from the fits in Figure 3a. Despite the relatively large error in determining BE_TX_ and BE_BX_ due to limited photon counts, aggregated data, presented in Figure 3b, give mean BE_TX_ and BE_BX_ of 1.13 ± 0.27 meV and 0.46 ± 0.28 meV, respectively, in good agreement with the mean BE_BX_ of 0.70 ± 0.23 meV extracted from pair detections. This shows again the validity of our approach and demonstrates that multiple excitons attractively interact in these QDs despite weak quantum confinement. It should be noted that the TX state involves excitation of the p-state, which has a higher energy than that of the s-state involved in band-edge excitation. Nevertheless, since the TX can relax either via emission from the s-state or via emission from the p-state, the higher energy of the p-state does not necessarily reflect on the BE_TX_. In CdSe and core–shell CdSe/CdS QDs, for example, it was found that s-state emission completely dominated TX emission.^8,9^ From our measurements, it appears that the same occurs for CsPbBr_3_.

**Figure 3:**
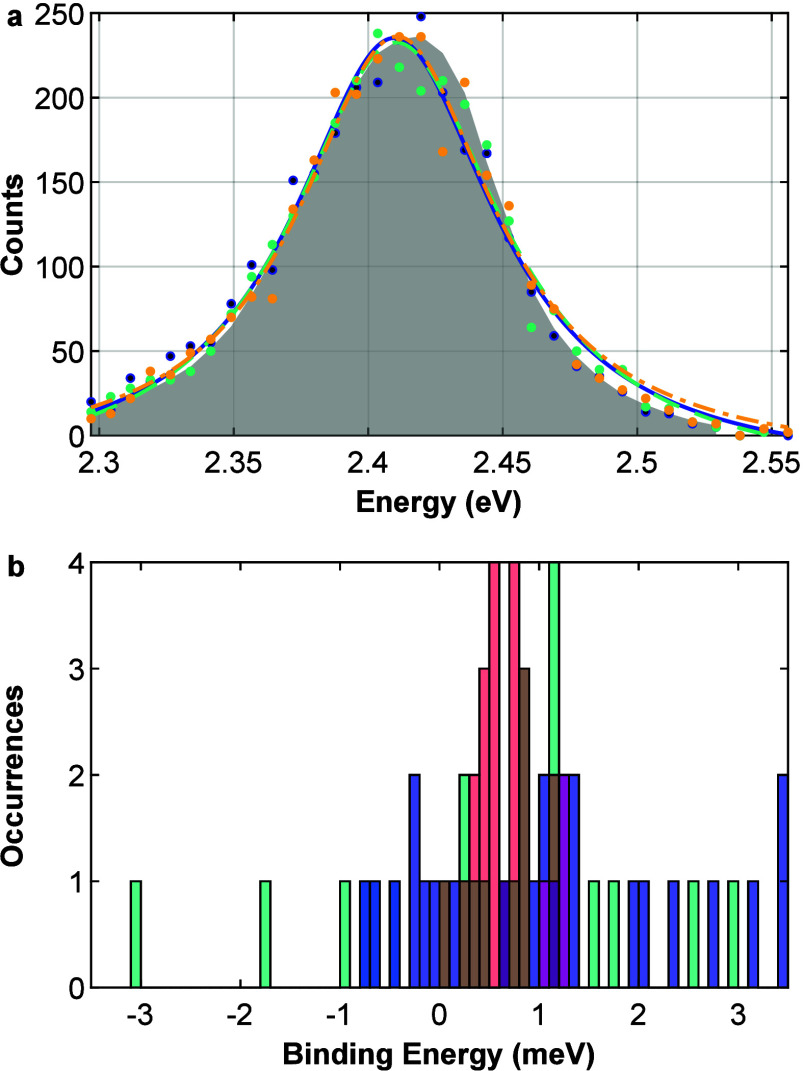
(a) Triple-cascaded photons from a single measurement, time-gated, and plotted as a function of their detection energy. TX, BX, and 1X photons marked in blue, green, and yellow dots, respectively. The obtained spectra are then fitted using a Voigt function (lines), respectively. Gray area corresponds to all photons detected without time gating, scaled. (b) Ensemble histogram of the binding energies calculated from the Voigt fits in (a) for BE_TX_ and BE_BX_, with blue and green bars, respectively. BE_BX_ calculated from pair-photon analysis is plotted in red bars.

In QDs composed of a single material or in type-I QDs, where all charge carriers are confined to the same volume, the interaction between multiple excitations is typically attractive and stronger than in the bulk due to the correlation energy of confined excitons. Previous measurements of 6 nm CsPbBr_3_ QDs featured an attractive BX interaction (BE_BX_ = 10 ± 6 meV) at room temperature and clear correlation of the BE_BX_ with charge-carrier confinement.^14,25^ Here, the large size of the CsPbBr_3_ QDs places them in the weak-confinement regime, having a diameter almost 4 times larger than the exciton Bohr diameter of 7 nm.^16^ This can explain the very low BEs measured (notably, the values measured here are significantly lower than those measured at cryogenic temperatures, which may indicate the role of dynamic disorder in modifying multiexciton interactions).^22^ In fact, the measured BE_BX_ is in good agreement with the extrapolated value from the emission-wavelength dependence of BE_BX_ in smaller CsPbBr_3_ QDs.^14^ We also note that the BE_BX_ extracted from the pair analysis shows a clear trend of higher BE_BX_ with higher energy of the 1X transition (indicating a stronger confinement and a smaller size, see Figure S4), in agreement with previously measured trends for stronger confinement.^14^

In weakly confined nanocrystals, Auger recombination is not an effective nonradiative decay mechanism.^20,26,27^ This implies that the second-order correlation at a time-delay of zero, g^(2)^(0), is likely close to 1. Indeed, this is observed here, as shown for a representative particle in Figure 4a, for which we get g^(2)^(0) = 0.98 ± 0.002 (see details in Supporting Information section S4). Thus, unlike the typical case of single-QD spectroscopy, where the hallmark of a single-particle emitter is a g^(2)^(0) < 0.5, here the antibunching value cannot be used as a signature of a single weakly confined QD.

**Figure 4:**
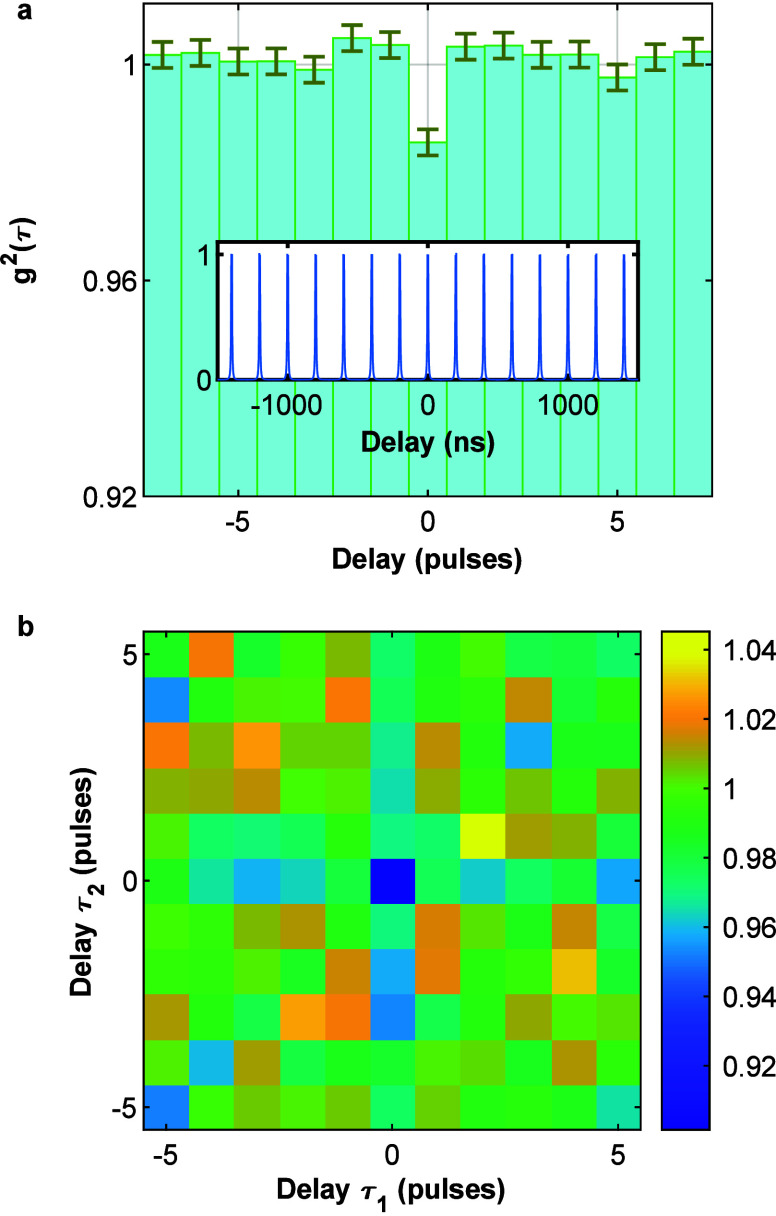
(a) Second-order correlation g^(2)^(τ), binned into intervals of the laser pulse period (200 ns). Inset: g^(2)^(τ) binned into finer intervals of 2.5 ns. (b) Third-order correlation g^(3)^(τ_1_,τ_2_), binned into intervals of the laser pulse period. All plots are normalized. Further information is given in Supporting Information sections S4 and S5.

To prove the observation of a single QD, one can observe that both the TX and the BX decay dynamics are nearly single-exponential (Figure S5). Had the source of emission been a pair of particles, there would have been two types of triple cascades—those where all three photons are emitted from one QD (exhibiting relatively faster dynamics) and those where two photons are emitted by one QD and another by the second QD (exhibiting relatively slower dynamics). This would result in biexponential decays for the TX and BX states, as we have recently shown in emission from uncoupled CdSe/CdS QD dimers.^28^

We further tested our triple-cascaded photon analysis by calculating the third-order photon correlation, g^(3)^(τ_1_,τ_2_). Such a plot is shown in Figure 4b and gives a g^(3)^(0,0) of 0.90 ± 0.015. This is calculated after filtering, counting only photon-detection events with a time gating of more than 0.2 ns and a spectral gating of more than 3 pixels between detections to reduce cross-talk (see details in Supporting Information section S5). Notably, the g^(2)^ calculated only from three photon detection events where one photon is delayed by up to 5 pulses from the other coinciding two (the diagonal and the *X*- and *Y*-axes in Figure 4b) gives a g^(2)^(0) value of 0.97 ± 0.003, in good agreement with a g^(2)^(0) of 0.98 ± 0.003 calculated for all double-cascaded events. Assuming that the three-body interaction is dominated by pairwise ones, one would expect that when g^(2)^(0) is close to unity, the deviation from unity of g^(3)^(0,0) is approximately three times that of g^(2)^(0).^7^ The measured values are commensurate with this.

Single QDs often exhibit blinking—most commonly expressed as correlated fluctuation of fluorescence intensity and lifetime, associated with trions and delayed emission.^29−31^ The triple-cascade photon measurement selectively probes the bright “on” state. Figure 5a presents the blinking trace of triple-photon detections (blue) and all photon detections (gray) for a characteristic QD, showing that the TX emission is produced almost exclusively from the bright “on” state. This allows for selective characterization specifically of photons emitted from the “on” state. This can also be observed from the ensemble 1X lifetimes extracted from the triple-cascade events, showing a mean τ_1X_ value of 3.33 ± 0.20 ns, in comparison to 4.30 ± 0.27 ns extracted from the biexponential fit of the lifetime plot of all detected photons (Figure 5b), which has a significantly large contribution from the longer lifetime typically associated with the “gray” state and with delayed emission (Figure S6). This also opens the opportunity to perform heralded spectroscopy on the “gray” state (or set of states) by selecting times when triple-photon events are absent. Interestingly, for the QD in Figure 5a, BX and TX emissions were suppressed to a larger degree than 1X emission during “off” periods. BX counts were suppressed by roughly a factor of 2 beyond 1X counts and TX counts by close to an order of magnitude (Figure S7). This was not observed in all nanocrystals and is an unexpected finding which needs further study, but may indicate potential routes toward strong reversible deviations from a Poisson distribution of the number of emitted photons, adjustable by the QD’s charge state.

**Figure 5:**
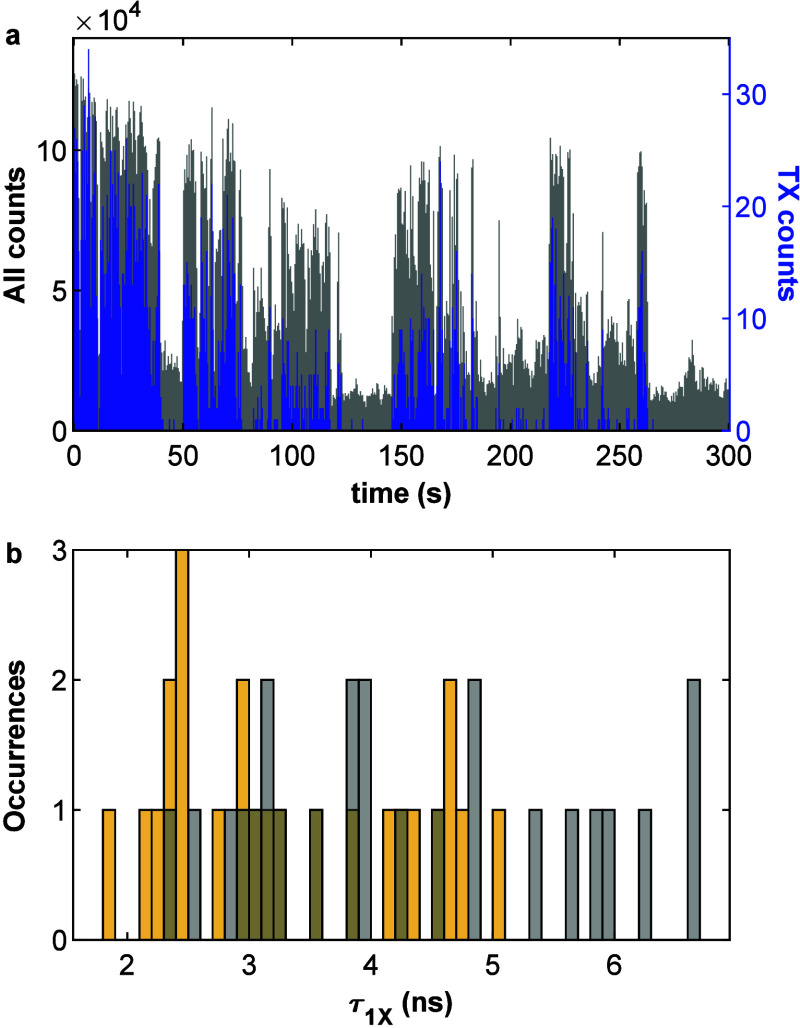
(a) Blinking trace of only triple-cascaded events in blue and from all detections in gray. Time bin: 500 ms. (b) Ensemble histogram of the 1X lifetime (τ_1X_) of triple-cascaded analysis and from all photon detections, yellow and gray, respectively.

In summary, we performed heralded spectroscopy on higher multiexcitonic states, opening a direct way to observe their spectroscopic properties. The ability to postselect desired events and hence specific emitting states is important for higher multiexcitons, where the signal is potentially convoluted with contributions from other states with similar kinetics. Our work also points at a way to identify single emitters when the commonly used criterion of g^(2)^(0) < 0.5 is violated. We do so by testing the temporal dynamics of the first arriving photons in a cascade for monoexponential behavior.^28^

For CsPbBr_3_ nanocrystals in the weakly confined regime, our results point to a large difference in the BX and TX binding energies at room temperature as compared with similarly sized particles at low temperature. One possible explanation for this relates to phonon-driven wavefunction localization at room temperature. Strong anharmonic lattice motion leads to a time-varying potential landscape for both electrons and holes, which can lead to partial localization of either of the two carriers comprising the exciton.^4,32,33^ This, in turn, reduces electron–hole overlap and thus significantly decreases BX (and higher multiexciton) binding energies in a manner similar to that for type-II or quasi type-II heterostructures.

Conceptually, the ability to generate and detect high-order multiexcitons points to a way for producing states of correlated photons with more than a pair of photons from a single QD in the weak-confinement regime. The average TX photon rate of ∼10 counts per second demonstrated here, which can be further increased with advances in monolithic SPAD array technology, is already very high compared to past demonstrations.^9,11^ Notably, multiphoton emission cascades, as presented here, are difficult to obtain without quantum-confined systems for two reasons. The first is that excitation of high carrier densities is achievable in a confined system by simply increasing the excitation power, as compared with bulk samples where plasma screening prohibits high excitation densities. The second is that level discretization provides us with a possible means to control the number of emitted photons and carrier interactions.

Finally, our ability to directly identify emission cascades, both in frequency and in time, and the very small values of the BX and TX binding energies, much less than the homogeneous line width, present interesting opportunities on the pathway to using emission cascades to obtain pairs or triplets of photons which go beyond classical time correlations if similar emission properties can be maintained at lower temperature and provided coherence can be maintained within the multiexciton emission lifetime.

## Supporting Information Available

QD synthesis, characterization and sample preparation, description of the experimental system, description of the data analysis of the triple cascaded photon measurements, description of the data analysis of g^(2)^ and g^(3)^ measurements, additional example of single particle measurements, and further triple-cascaded measurements analysis (PDF)
