# Supplementary material for: Observation of Three-Photon Cascaded Emission from Triexcitons in Giant CsPbBr3 Quantum Dots at Room Temperature
Source: Nano Lett. 2024 Oct 14;24(42):13185–91. doi: 10.1021/acs.nanolett.4c03096 (PMC11503816; doi:10.1021/acs.nanolett.4c03096)
Supplement: Supplementary file 1 — nl4c03096_si_001.pdf [file nl4c03096_si_001.pdf]

Supplementary Information

Observation of three-photon cascaded emission from triexcitons in giant $CsPbBr_3$ quantum dots at room temperature

Miri Kazes,[*,$] *Dekel Nakar,*[*,$] *Ihor Cherniukh,*[Δ,#] *Maryna I. Bodnarchuk,*[Δ,#] *Leon G. Feld,*[#,Δ,£] *Chenglian Zhu,*[#,Δ] *Daniel Amgar,*[*] *Gabriele Rainò,*[#,Δ,£] *Maksym V. Kovalenko,*[#,Δ,£] *Dan Oron*[*]

[*] Department of Molecular Chemistry and Materials Science, Weizmann Institute of Science, Rehovot 7610001, Israel

[Δ] Laboratory for Thin Films and Photovoltaics, Empa, Swiss Federal Laboratories for Materials Science and Technology, 8600 Dübendorf, Switzerland

[#] Institute of Inorganic Chemistry, Department of Chemistry and Applied Biosciences, ETH Zürich, 8093 Zürich, Switzerland

[£] National Centre of Competence in Research (NCCR) Catalysis, ETH Zürich, CH-8093 Zürich, Switzerland

[$] These authors contributed equally to this work

https://doi.org/10.1021/acs.nanolett.4c03096

S1. Quantum dot synthesis and sample preparation

Synthesis

The 26.5 nm NCs were synthesized by a modified TOPO-$PbBr_2$ approach[1] with a slow injection of Cs-OA and $PbBr_2$-TOPO precursors at higher temperature. The NCs were capped with ω-amino-terminated polystyrene (P18806-SNH2, Polymer Source Inc.), precipitated with hexane, dispersed in toluene, and further treated with 2-Ammonioethyl 2-octyl-1-dodecyl phosphate, which was synthesized following ref. 2.

Sample preparation

Samples of isolated nanocrystals were prepared by spin coating a dilute solution of the NCs dispersed in a 3 wt. % solution of polystyrene in anhydrous toluene on a glass coverslip.

S2. *Spectro*SPAD system details

The *spectro*SPAD is a spectrometer based on a monolithic linear array of single-photon avalanche diodes (SPADs), as described in ref.3 and ref.4. In this setup, a pulsed excitation laser (70 ps, 470 nm, 5 MHz, LDH-P-C-470B, PicoQuant) is focused on single particles on a glass cover slip using a standard inverted fluorescence microscope (Eclipse Ti-U, Nikon) with an immersion-oil objective (x100, 1.3 NA, Nikon). PL emission collected through the same objective is filtered using a dichroic mirror (FF484-FDi02-t3, Semrock) and a long-pass filter (BLP01-473R, Semrock). The image plane of the microscope serves as the input for a Czerny–Turner spectrometer composed of a 2f–2f system (AC254-300-A-ML and AC254-100-A-ML, Thorlabs) and a blazed grating (53-*-201R, Richardson Gratings). The output of the spectrometer is coupled to the SPAD array. The SPAD array consists of 512 pixels with a physical pixel pitch is 26.2 µm, amounting to ~1.7 nm wavelength resolution (7–11 meV) out of which 64 pixels are connected to 64 time-to-digital converters (TDCs) implemented by a field-programmable gate array (FPGA), which time-tags photon detection events. The temporal instrument response function (IRF) has ~180 ps full-width at half maximum (FWHM). One of the pixels has a very high dark count rate and was excluded from the analysis.

S3. Data Analysis of triple-cascaded photon measurements

The stream of photons are tagged according to their arrival time from the start of the measurement (in units of the instrument time resolution, ~17.9 ps) and to the detection channel. We translate detection times to delay times by modulo operation, i.e., remainder by division of the detection time stamps by the laser period (200 ns) in units of the instrument time resolution (after shifting the times to coincide with the peak of the laser, found by the peak intensity). In order to find tripe-cascaded photon events, we identify in our data detection events in which three photons are detected within the same pulse. Within each triplet we identify the first, second, and third detected photons. Delay times for the first photon are calculated from the peak of the laser, and delay times of subsequent photons are calculated from the detection of the previous photon.

For the delay time histogram, we group the detection delay times into bins of 250 ps and form a histogram for each of the first, second, and third detected photon.

S4. Second-order correction function calculation

The second-order correlation function, $g^{(2)}(\tau)$, measures the normalized probability to detect two photons as a function of the delay time τ between them. $g^{(2)}(0)$ is the probability to detect two photons following the same excitation pulse, normalized to the probability to detect two photons, each following a different excitation pulse. Analysis is done based on the procedure in refs. 5,6, with the following details. First we find all photon pairs with up to 7.5 laser periods of delay between them (1500 ns), and that were detected by different detector channels (to exclude the effect of detector-channel dead-time). We take the photon pairs detected in our 63 low-noise channels and randomly assign the photons in each pair to the "first" and "second" artificial channels. We then calculated the delay time τ between the detections in the "first" and "second" artificial channels. The detection events are binned to 2.5 ns delay-time resolution for smoothing. We reduce the predicted cross-talk and dark-count contributions based on their measured average probabilities. The datapoints at delays of whole multiples of the laser period are excluded since the estimation of the cross-talk contribution at zero delay is not accurate enough and introduces excessive noise. To generate a double-sided plot, the counts in the one-sided correlation function are split into positive and negative delays according to the binomial distribution.

The high-resolution graph in the inset to Fig. 4a is a plot of this one-dimensional data after normalizing by division by the mean peak value for the side peaks ($G^{(2)}(\infty)$). The graph in Fig. 4a is calculated by binning the data to the pulse-period delay-time resolution: The size of the central peak ($G^{(2)}(0)$) is the area under the curve for absolute delays of up to half the laser period (±100 ns). It is then normalized by dividing by the average area of the side peaks ($G^{(2)}(\infty)$), yielding the herein-reported value of $g^{(2)}(0) = G^{(2)}(0) / G^{(2)}(\infty)$. The error bars in Fig. 4a (0.24%) are the standard deviation of the values of the different side peaks (calculated for n=40 side peaks from the double-sided graph), normalized identically.

S5. Third-order correlation function calculation

The third-order correlation function, $g^{(3)}(\tau_1,\tau_2)$, measures the normalized probability to detect three photons as a function of the delay times τ_1 and τ_2 between them. Here, delay times are in multiples of the laser period (200 ns), and $g^{(3)}(0,0)$ is the probability to detect three photons following the same excitation pulse, normalized to the probability to detect three photons, each following a different excitation pulse. We calculate it by finding all photon triples with up to 5 laser periods of delay between them, and that were detected by different detector channels (to exclude the effect of detector channel dead-time). We take the photon triplets detected in our 63 low-noise channels, and (similarly to our

generation of the two-sided $g^{(2)}(\tau)$ graph), randomly assign the photons in each triplet to the "first", "second", and "third" artificial channels. We then calculated the delay times $\tau_1 = t_2 - t_1$ and $\tau_2 = t_3 - t_2$, where t_1, t_2, and t_3 are the laser-cycle indices of the detections in the "first", "second", and "third" artificial channels. To mitigate the noise of cross-talk between detector channels, only photon detection events with a time gate of more than 0.2 ns and a spectral gate of more than 3 pixels between detections were used. The two-dimensional plot of triplet counts $[G^{(3)}(\tau_1,\tau_2)]$ was normalized to produce $g^{(3)}(\tau_1,\tau_2)$ by dividing by the average of the counts in all 90 time-bins for which t_1, t_2, and t_3 are different (i.e., $\tau_1 \neq 0$, $\tau_2 \neq 0$, and $\tau_1 \neq \tau_2$), meaning not on the diagonal and central x and y axes. The standard deviation of these bins (1.5%) was used also as the standard deviation for $G^{(3)}(0,0)$. Common error propagation was used.

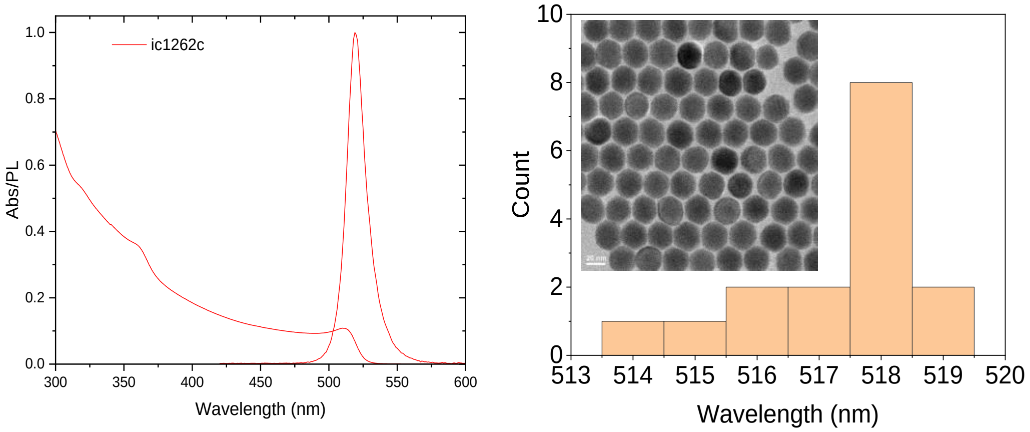

Figure S1. $CsPbBr_3$ QDs in solution. a) Absorption and emission spectra of giant CsPbBr3 QDs in solution. b) Size distribution presented by a histogram of the emission peak position. Inset: TEM image with a scale bar of 20nm.

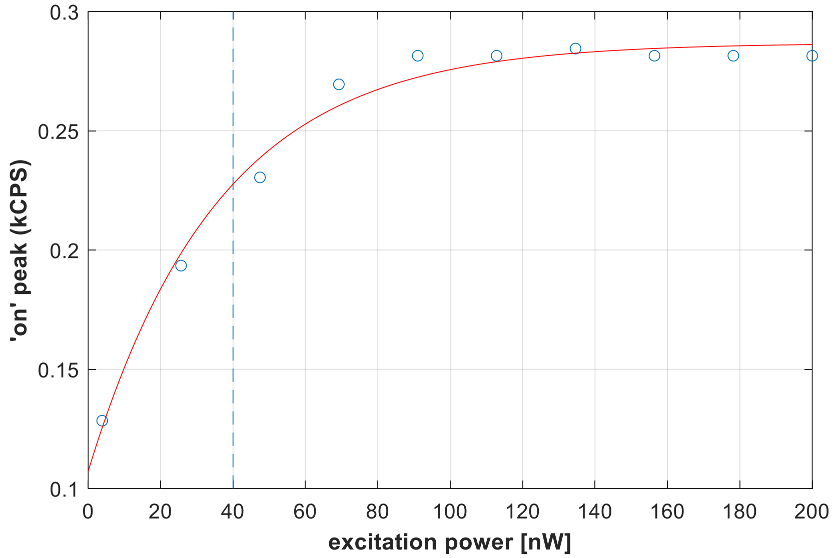

Figure S2. Saturation curve measured on a single $CsPbBr_3$ particle. Excitation power of 40 nW was used in the TX measurements (dotted blue line). The calculated saturation power from the fit (red line) is 36 ±9 nW.

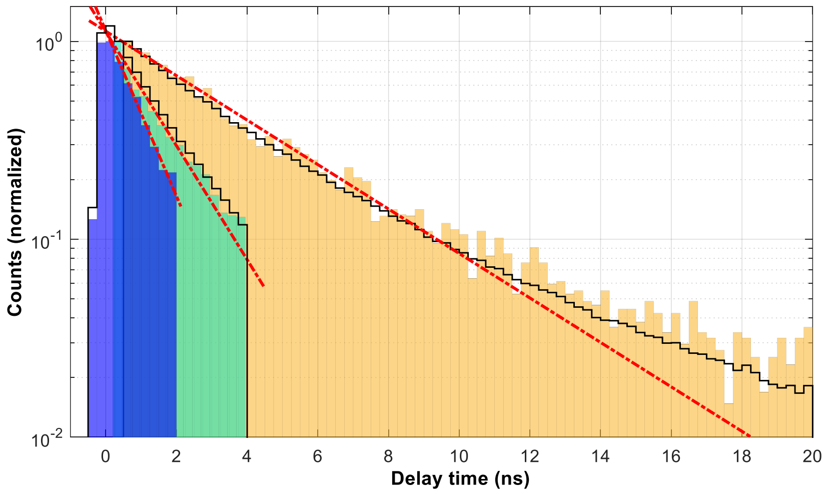

Figure S3. Histogram of triple-cascaded photons from another single-particle measurement, time gated and plotted according to their arrival time. TX, BX, and 1X photons marked in blue, green, and yellow bars, respectively. Red lines correspond to mono-exponential decay fits. Black lines correspond to histograms taken from two-photon analysis.

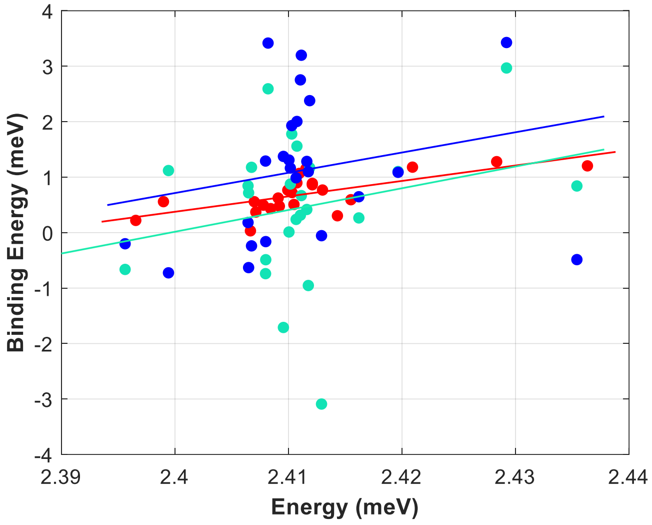

Figure S4. Correlation of the binding energy with the 1X emission peak, from the 24 particles analyzed in this work. BE_{BX} extracted from bi-herald analysis, in red, gives a correlation coefficient of 0.68. BE_{BX} and BE_{TX} extracted from tri-herald analysis show very weak correlation coefficients of ~0.25, in green and blue, respectively.

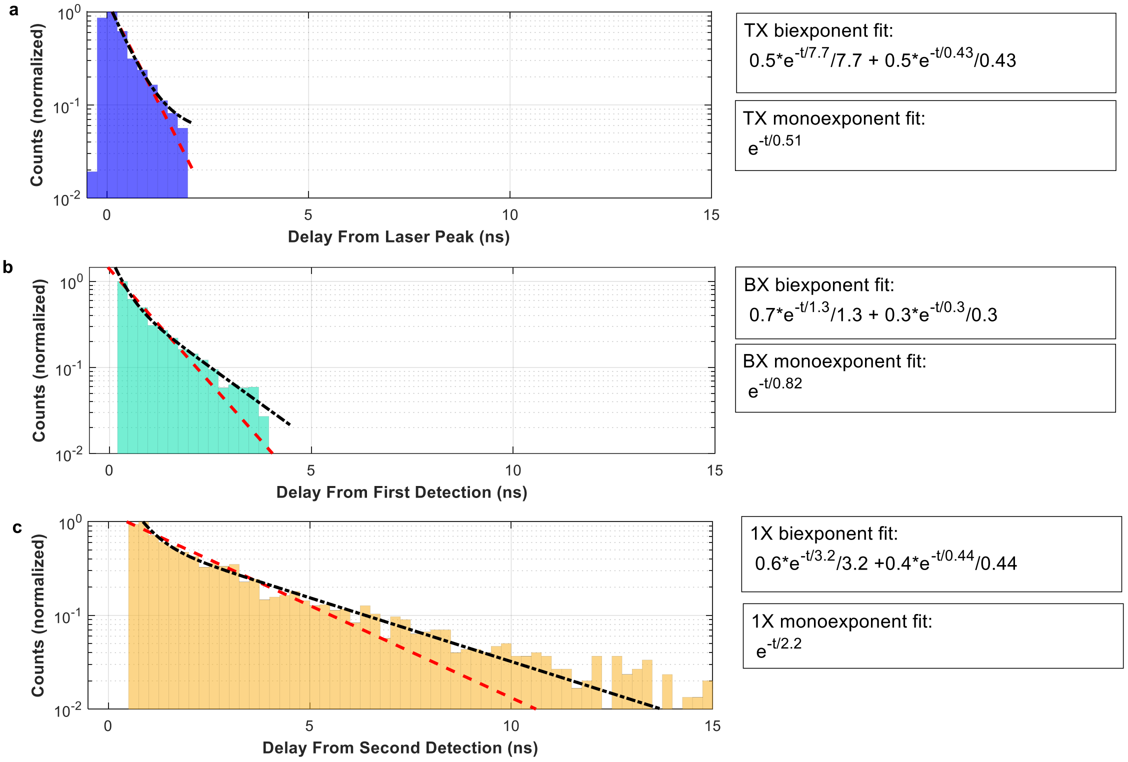

Figure S5. Histogram of (a) TX, (b) BX and (c) 1X detection times from triple-cascaded events, along with a mono- and a bi-exponential decay fit, red and black lines, respectively, for the particle shown in Figure 2(a).

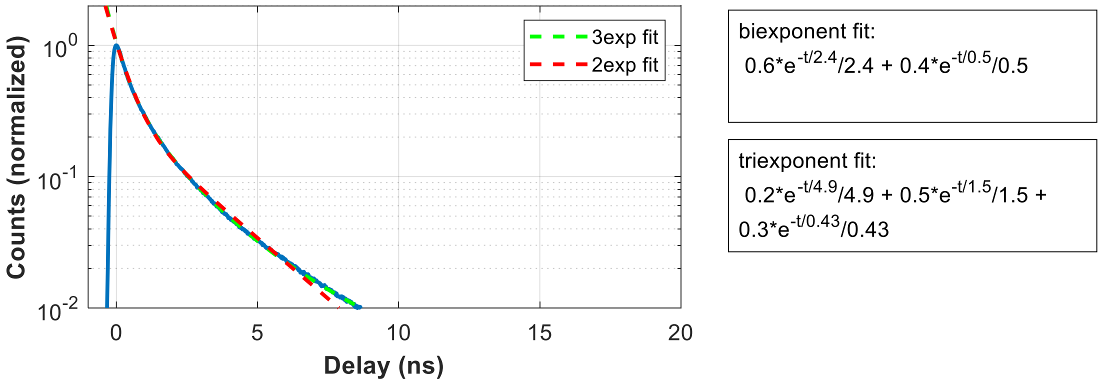

Figure S6. Lifetime plot of all detected photon (blue line) along with a bi-exponential and tri-exponential decay fits, red and green dashed lines, respectively, for the particle shown in Figure 2(a).

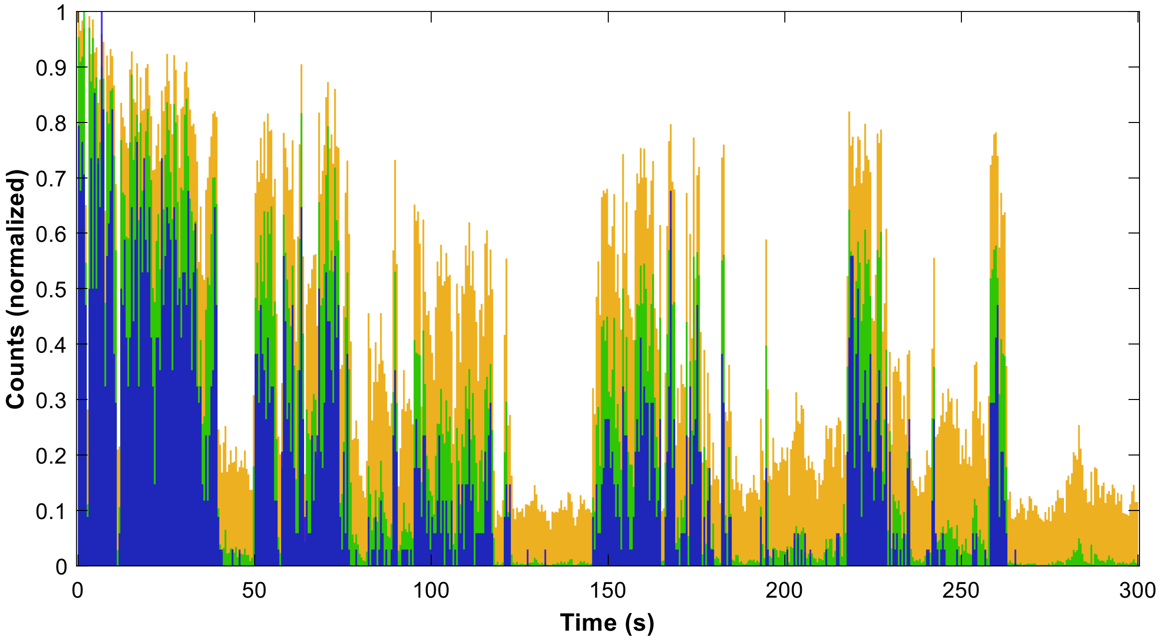

Figure S7. Blinking trace of all detected photons (orange), double-cascaded events (green), and triple-cascaded events (blue), normalized, for the particle shown in Figure 2(a). Time bin: 500 ms.

References

(1) Akkerman, Q. A.; Nguyen, T. P. T.; Boehme, S. C.; Montanarella, F.; Dirin, D. N.; Wechsler, P.; Beiglböck, F.; Rainò, G.; Erni, R.; Katan, C.; Even, J.; Kovalenko, M. V. Controlling the Nucleation and Growth Kinetics of Lead Halide Perovskite Quantum Dots. *Science (80-.).* **2022**, *377* (6613), 1406–1412. https://doi.org/10.1126/science.abq3616.

(2) Morad, V.; Stelmakh, A.; Svyrydenko, M.; Feld, L. G.; Boehme, S. C.; Aebli, M.; Affolter, J.; Kaul, C. J.; Schrenker, N. J.; Bals, S.; Sahin, Y.; Dirin, D. N.; Cherniukh, I.; Raino, G.; Baumketner, A.; Kovalenko, M. V. Designer Phospholipid Capping Ligands for Soft Metal Halide Nanocrystals. *Nature* **2023**, *626*. https://doi.org/10.1038/s41586-023-06932-6.

(3) Lubin, G.; Yaniv, G.; Kazes, M.; Ulku, A. C.; Antolovic, I. M.; Burri, S.; Bruschini, C.; Charbon, E.; Yallapragada, V. J.; Oron, D. Resolving the Controversy in Biexciton Binding Energy of Cesium Lead Halide Perovskite Nanocrystals through Heralded Single-Particle Spectroscopy. *ACS Nano* **2021**, *15* (12), 19581–19587. https://doi.org/10.1021/acsnano.1c06624.

(4) Lubin, G.; Tenne, R.; Ulku, A. C.; Antolovic, I. M.; Burri, S.; Karg, S.; Yallapragada, V. J.; Bruschini, C.; Charbon, E.; Oron, D. Heralded Spectroscopy Reveals Exciton-Exciton Correlations in Single Colloidal Quantum Dots. *Nano Lett.* **2021**, *21* (16), 6756–6763. https://doi.org/10.1021/acs.nanolett.1c01291.

(5) Frenkel, N.; Scharf, E.; Lubin, G.; Levi, A.; Panfil, Y. E.; Ossia, Y.; Planelles, J.; Climente, J. I.; Banin, U.; Oron, D. Two Biexciton Types Coexisting in Coupled Quantum Dot Molecules. *ACS Nano* **2023**, *17* (15), 14990–15000. https://doi.org/10.1021/acsnano.3c03921.

(6) Lubin, G.; Tenne, R.; Michel Antolovic, I.; Charbon, E.; Bruschini, C.; Oron, D. Quantum Correlation Measurement with Single Photon Avalanche Diode Arrays. *Opt. Express* **2019**, *27* (23), 32863. https://doi.org/10.1364/OE.27.032863.
